# Pathways to post‐traumatic stress disorder and alcohol dependence: Trauma, executive functioning, and family history of alcoholism in adolescents and young adults

**DOI:** 10.1002/brb3.1789

**Published:** 2020-09-29

**Authors:** Stacey Subbie‐Saenz de Viteri, Ashwini Pandey, Gayathri Pandey, Chella Kamarajan, Rebecca Smith, Andrey Anokhin, Lance Bauer, Annah Bender, Grace Chan, Danielle Dick, Howard Edenberg, Sivan Kinreich, John Kramer, Marc Schuckit, Yong Zang, Vivia McCutcheon, Kathleen Bucholz, Bernice Porjesz, Jacquelyn L. Meyers

**Affiliations:** ^1^ State University of New York Downstate Health Sciences University Brooklyn New York USA; ^2^ Virginia Commonwealth University Richmond Virginia USA; ^3^ Washington University School of Medicine St. Louis Missouri USA; ^4^ University of Connecticut School of Medicine Farmington Connecticut USA; ^5^ University of Missouri St. Louis Missouri USA; ^6^ Indiana University School of Medicine Indianapolis Indiana USA; ^7^ University of Iowa Iowa City, Iowa USA; ^8^ University of California San Diego California USA

**Keywords:** alcoholism, executive function, post‐traumatic stress disorder, trauma

## Abstract

**Introduction:**

Family history (FH) of alcohol dependence is likely to increase the risk of trauma exposure, post‐traumatic stress disorder (PTSD), and alcohol dependence. FH of alcohol dependence and trauma has been separately shown to adversely affect planning/problem‐solving aspects of executive function. However, few studies have examined these risk factors in an integrated model.

**Methods:**

Using data from trauma‐exposed individuals from the Collaborative Study on the Genetics of Alcoholism prospective cohort (*N* = 1,860), comprising offspring from alcohol‐dependent high‐risk and comparison families (mean age [*SE*] = 21.9 [4.2]), we investigated associations of trauma (nonsexual assaultive, nonassaultive, sexual assaultive) with DSM‐IV PTSD and alcohol dependence symptom counts, and planning/problem‐solving abilities assessed using the Tower of London Test (TOLT). Moderating effects of family history density of alcohol use disorder (FHD) on these associations and sex differences were explored.

**Results:**

Family history density was positively associated with PTSD in female participants who endorsed a sexual assaultive trauma. Exposure to nonsexual assaultive trauma was associated with more excess moves made on the TOLT.

**Conclusion:**

Findings from this study demonstrate associations with PTSD and alcohol dependence symptom counts, as well as poor problem‐solving ability in trauma‐exposed individuals from families densely affected with alcohol dependence, depending on trauma type, FHD, and sex. This suggests that having a FH of alcohol dependence and exposure to trauma during adolescence may be associated with more PTSD and alcohol dependence symptoms, and poor problem‐solving abilities in adulthood.

## Graphical abstract

Findings from this study demonstrate associations with PTSD and alcohol dependence symptom counts, as well as poor problem‐solving ability in trauma‐exposed individuals from families densely affected with alcohol dependence, depending on trauma type, FH density of alcoholism, and sex. This suggests that having a FH of alcohol dependence and exposure to trauma during adolescence may be associated with more PTSD and alcohol dependence symptoms, and poor problem‐solving abilities in adulthood.

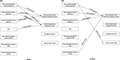

## 1 INTRODUCTION

Exposure to a traumatic event, such as a physical or sexual assault, or witnessing violence, can have many adverse consequences, including subsequent post‐traumatic stress disorder (PTSD) increased alcohol misuse (Breslau, 2009; Breslau, Troost, Bohnert, & Luo, 2013; Keyes, Hatzenbuehler, & Hasin, 2011) and poorer cognitive functioning (Aupperle, Melrose, Stein, & Paulus, 2012; Flaks et al., 2014; Olff, Polak, Witteveen, & Denys, 2014). It is estimated that 90% of the United States population are exposed to some type of trauma (Kilpatrick et al., 2013). However, only 6%–8% of the population are diagnosed with PTSD (Breslau, 2009; Ozer, Best, Lipsey, & Weiss, 2003). DSM‐IV alcohol dependence is more prevalent than PTSD, with approximately 12%–18% of the population meeting diagnostic criteria during their lifetime (Blanco et al., 2013; Hasin, Stinson, Ogburn, & Grant, 2007). Further, PTSD has high comorbidity with alcohol dependence (Blanco et al., 2013; Kessler, Chiu, Demler, Merikangas, & Walters, 2005).

The type of trauma to which one is exposed can influence the adverse consequences experienced (Ozer et al., 2003). Traumatic events are often considered in two broad categories: assaultive and nonassaultive (Cisler et al., 2011; Resnick, Kilpatrick, Dansky, Saunders, & Best, 1993). Assaultive trauma can be further broken down into whether or not it is sexual in nature. For example, individuals exposed to nonsexual or sexual assaultive trauma are more likely to develop PTSD compared to those exposed to nonassaultive trauma (Cisler et al., 2011; Kessler et al., 2005; Ozer et al., 2003; Resnick et al., 1993). While many studies examine nonsexual and sexual assaultive trauma together as assaultive trauma, it may be important to separate these two trauma types. For example, Breslau et al. (1998) showed that the highest risk of PTSD was associated with physical assaultive violence, while Lang et al. (2003) showed that sexual assault was associated with increased substance use (Breslau et al., 1998; Lang et al., 2003). Importantly, trauma type may also moderate the association between PTSD and alcohol dependence. For example, some studies found that individuals exposed to nonassaultive trauma were more likely to initiate substance use earlier compared to those with an assaultive trauma history (Bucholz et al., 2017), while other studies found that assaultive trauma, but not nonassaultive trauma, exposure was associated with increases in binge drinking (Cisler et al., 2011). Thus, consideration of trauma type is critical in better understanding potential adverse consequences following trauma exposure.

There are known sex differences in the rates of trauma exposure, as well as PTSD and alcohol dependence diagnoses (Beals et al., 2013; Boudoukha, Ouagazzal, & Goutaudier, 2017; Chung & Breslau, 2008; Erol & Karpyak, 2015). For example, men are more likely to be exposed to trauma during their lifetime, but less likely to meet criteria for PTSD compared to women (Kessler, Sonnega, Bromet, Hughes, & Nelson, 1995). This could potentially be due to the increased likelihood of women experiencing recurring, high impact traumatic exposures (e.g., childhood sexual assault, physical abuse) (Hien, Cohen, & Campbell, 2005). Further, women with lifetime alcohol dependence are more likely to report lifetime PTSD than men (Kessler et al., 1997). These findings further underscore the need to consider the role of trauma type as well as sex when examining the relationship between PTSD and alcohol dependence.

Given the common comorbidity of PTSD and alcohol dependence (Allen, Crawford, & Kudler, 2016; Goldstein, Bradley, Ressler, & Powers, 2017; Kessler et al., 2005), researchers have proposed several hypotheses linking the two disorders. One common theory is the self‐medication hypothesis, which suggests that individuals suffering from PTSD symptoms self‐medicate with alcohol and subsequently develop an alcohol use problem (Allen et al., 2016; Goldstein et al., 2017). Another hypothesis is the shared‐liability model, which suggests that PTSD and alcohol dependence have shared risk factors (Allen et al., 2016; Goldstein et al., 2017), such as having a family history (FH) of alcohol dependence, neurocognitive and behavioral impairments, and genetic risk. This study used an integrated model to investigate the associations of different types of trauma exposure and FH of AUD on PTSD and/or alcohol dependence as well as planning and problem‐solving aspects of executive function, while also investigating sex differences.

Individuals with a FH of alcohol dependence have been shown to have disproportionately high rates of trauma exposure and subsequent PTSD compared to individuals without a FH of dependence. For example, one study demonstrated that mother's alcohol dependence status was positively associated with offspring PTSD (Bender, Meyers, McCutcheon, & Bucholz, 2016). This may be due to adverse aspects of the home environment, like having an abusive or neglectful parent or relative (Breslau et al., 2013), as previous studies have demonstrated that having parents with alcohol use problems increases risk for exposure to a traumatic event (Breslau et al., 2013; Dube et al., 2001; Meyers et al., 2014). Further, individuals with a FH of alcohol dependence are at risk, not just because of their environment, but also because of their genetic risk for alcohol dependence, which shares a substantial overlap in genetic influences with PTSD. Studies have shown that genetic influences on PTSD may be around 30%–40% (Banerjee, Morrison, & Ressler, 2017; Nugent, Amstadter, & Koenen, 2008; Stein, Jang, Taylor, Vernon, & Livesley, 2002; True et al., 1993) and alcohol dependence around 50%–60% (Reilly, Noronha, Goldman, & Koob, 2017; Walters et al., 2018). Further, Sartor et al. showed that PTSD and alcohol dependence had a genetic correlation of 0.54 (Sartor et al., 2011). In addition, a recent study by Sheerin et al. on shared molecular genetic basis for alcohol dependence and PTSD showed lower heritability for PTSD and alcohol dependence (*h* ^2^ = 0.18 and 0.09, respectively) and a significant correlation between alcohol dependence and PTSD (*r* = .35) and a moderate, but significant correlation between alcohol dependence and PTSD (*r* = .34) in female participants (Sheerin et al., 2020). Although there are environmental and genetic contributions to both PTSD and alcohol dependence, most studies only examine dichotomous parental alcohol dependence status. A family history density (FHD) measure is useful for disorders influenced by genetic and environmental components, such as PTSD and alcohol dependence, since it can include other affected family members and account for degrees of relatedness (Cservenka, Gillespie, Michael, & Nagel, 2015; Pandey et al., 2020; Powers, Berger, Fuhrmann, & Fendrich, 2017).

Post‐traumatic stress disorder and alcohol dependence also share behavioral impairments, including hyperarousal, irritability, and reckless behavior, which are thought to be due to deficits in executive functions (Koob & Volkow, 2016; Logrip, Zorrilla, & Koob, 2012). Executive functions are central in carrying out efficient adaptive and goal‐directed behaviors such as inhibition and attentional control, set‐shifting and mental flexibility, working memory, and planning and problem‐solving (Packwood, Hodgetts, & Tremblay, 2011; Pennington & Ozonoff, 1996). While there are many studies investigating associations between PTSD and aspects of executive functioning (Flaks et al., 2014; Olff et al., 2014), one aspect of executive functioning, planning, and problem‐solving abilities have been understudied in individuals with PTSD. In the few studies that have been conducted (Aupperle et al., 2012) results were inconsistent, limited by small sample sizes of individuals with or without PTSD and only a single study investigated a specific type of trauma (Kanagaratnam & Asbjornsen, 2007; Lagarde, Doyon, & Brunet, 2010; Twamley et al., 2009; Vasterling et al., 2002). This suggests that the relationship between trauma, PTSD, and planning and problem‐solving needs further investigation in larger samples of male and female participants.

One task that has been used to assess planning and problem‐solving aspects of executive functioning is the Tower of London test (TOLT) (Ruocco et al., 2014). Although activation of different brain areas has been observed depending on the different versions of the TOLT, one area consistently activated is the prefrontal cortex, which plays a significant role in executive function by monitoring activities in other cortical and subcortical regions (Baker et al., 1996; Ruocco et al., 2014). Therefore, executive dysfunction in PTSD and alcohol dependence may be partly due to the shared neurocircuitry of PTSD and addiction, involving hypoactivation of prefrontal regions (Aupperle et al., 2012; Koob & Volkow, 2016). A particularly vulnerable period for prefrontal development is adolescence, when the brain undergoes important developmental changes, including synaptic pruning in this region (Juraska & Willing, 2017; Laviola & Marco, 2011). This period also coincides with peak alcohol experimentation shown to impact brain development (Koob & Volkow, 2016). In part, this may contribute to why trauma experienced during childhood and adolescence has particularly adverse and enduring consequences on the individual's mental health and cognitive functioning (Dube et al., 2003; Hussey, Chang, & Kotch, 2006; Springer, Sheridan, Kuo, & Carnes, 2007). These consequences have been shown to persist throughout the individual's lifespan, increasing risk for mental health problems, including PTSD and alcohol misuse (Follette, Polusny, Bechtle, & Naugle, 1996; Green et al., 2000; Khoury, Tang, Bradley, Cubells, & Ressler, 2010) Further, models of AUD risk differ for adolescents compared to adults (Fowler et al., 2007). For example, heritability estimates, and the relative importance of genetic and environment risk factors change across adolescence and emerging adulthood (Edwards et al., 2017; Edwards & Kendler, 2013), with research demonstrating that the specific social‐environmental factors impacting alcohol misuse in adolescence (e.g., access, parental monitoring, peer substance use) differ adulthood.

It is possible that trauma type and gender may also play a role in the relationship between trauma, FH of AUD, PTSD, and executive function. One study by Meyers et al. (2019) showed neurocognitive differences depending on trauma type, such that sexual assaultive trauma, and not nonsexual assaultive or nonassaultive trauma, was associated with decreased rate of change in frontal theta oscillations during response inhibition, particularly among female participants (Meyers et al., 2019). This study suggests that trauma type, specifically sexual assaultive trauma, may affect executive functioning differently among female individuals compared to other trauma types. Further, another study examined the impact of fathers’ alcohol problems and showed that early adolescent boys, but not girls, had poorer performance on a TOLT than boys from control families (Adkison et al., 2013), suggesting sex differences in the association of paternal alcohol use disorder (AUD) with executive function deficits during early adolescence. However, no prior study has examined neurocognitive risk factors for PTSD among male and female adolescents with a FH of alcohol dependence leaving important questions regarding any potential neurocognitive or behavioral deficits associated with trauma experienced among the offspring of alcoholics unanswered.

In the current study, we investigated the influence of FH of AUD, operationalized as FHD, on the associations between trauma type (nonsexual assaultive, nonassaultive, and sexual assaultive), and PTSD/alcohol dependence symptom counts, as well as planning and problem‐solving aspects of executive functioning using an integrated model in trauma‐exposed individuals from the Collaborative Study on the Genetics of Alcoholism (COGA) prospective cohort (*N* = 1,860). Secondary analyses included sex‐stratified models. We hypothesized that FHD would moderate these associations depending on the trauma type, such that as FHD increases individuals with high impact traumatic exposures (i.e., sexual assaultive trauma) will have more PTSD symptoms, and that this will also vary by sex since women are more likely to experience sexual assaultive trauma than men.

## 2 METHODS

### 2.1 Sample

This study utilized data from the COGA prospective study, which has been ongoing since 2004. Details on data collection and procedures have previously been published (Bucholz et al., 2017). The COGA prospective study comprises offspring, both with and without alcohol use problems, from alcohol‐dependent high‐risk and community comparison families who were 12–22 years of age at enrollment and have had multimodal assessments (clinical, behavioral, neuropsychological, neurophysiological) approximately every 2 years. For the purpose of this cross‐sectional study, data from the last interview were used for each individual. Only trauma‐exposed individuals (*N* = 1,860) were included in this study since exposure to a traumatic event had to be present in order for an individual to be asked questions concerning PTSD symptoms. The sample was 47.1% female with a mean age of 21.9 (*SD* = 4.2) at the most recent interview.

### 2.2 Ethical statement

Informed consent was obtained from each participant, and experimental protocols were in accordance with US Federal Policy of protection of human subjects ethical guidelines and approved by each site's Institutional Review Board. Parental permission was obtained for all participants under the age of 18.

### 2.3 Measures

#### 2.3.1 Trauma exposure and DSM‐IV PTSD and alcohol dependence symptom counts

Trauma exposure was defined using the Semi‐Structured Assessment for the Genetics of Alcoholism (SSAGA‐IV) interview, which consists of questions regarding potentially traumatic life events involving physical and sexual violence, witnessing violence, and witnessing natural disasters and accidents. Consistent with previous studies (Bender et al., 2016; McCutcheon et al., 2017; Meyers et al., 2019), three types of traumatic exposures were considered: nonsexual assaultive, nonassaultive, and sexual assaultive trauma. These were lifetime binary variables (0 = no; 1 = yes). Nonsexual assaultive trauma was defined as having been shot, stabbed, mugged or threatened with a weapon, kidnaped, or held captive and/or tortured. Nonassaultive trauma was defined as unexpectedly discovering a dead body, experiencing a natural disaster, or experiencing a life‐threatening accident. Sexual assaultive trauma was defined as being raped or otherwise sexually assaulted by a relative or nonrelative. DSM‐IV PTSD symptom counts were recorded if an individual endorsed one or more traumatic experiences. A maximum endorsement of 17 symptoms were possible in for the three PTSD symptom criteria defined by the DSM‐IV: five re‐experiencing symptoms, seven avoidance symptoms, and five arousal symptoms (American Psychiatric Association, 2000). Individuals could endorse a maximum of seven symptoms for DSM‐IV alcohol dependence. As only data on DSM‐IV PTSD were available, DSM‐IV alcohol dependence was used to remain consistent for both PTSD and alcohol dependence.

#### 2.3.2 Family history density of AUDs (FHD)

Family history of DSM‐5 AUD was ascertained for adult subjects and adult family members through the SSAGA, and adolescents through the child and adolescent tailored CSSAGA (Bucholz et al., 1994). A FHD ratio was calculated for each individual from both alcohol and community control families, with reported information for four or more relatives (*N* = 1,704). We note that while those from community comparison families had lower FHD scores on average, compared with those ascertained from families densely affected with alcohol dependence (mean FHD[*SD*]: 0.299[0.152] vs. 0.513[0.154]), many had scores > 0 and all participants were included in these analyses. Details regarding the calculation of FHD can be found in Pandey et al. (2020). Briefly, affectedness was defined as the individual's first‐ and second‐degree relatives’ (parents, grandparents, parental siblings, full siblings, half‐siblings) AUD symptom count values divided by the maximum possible value of 1 (range from 0 to 1). Weighting was applied, such that the weight decreases with the increase in degree of relatedness. This measure, referred to as FHD in this study, has been found to be a sensitive indicator of individuals with DSM‐IV alcohol dependence (Pandey et al., 2020).

#### 2.3.3 Tower of London test

A computerized version of the TOLT was administered using the Colorado assessment tests (CATs) for cognitive and neuropsychological assessment (Davis, 2002). The TOLT was used to assess planning and problem‐solving skills (Shallice, 1982) and has been described in a previous publication (Pandey et al., 2018). Briefly, participants were required to move colored beads on a computer screen from a starting position to a goal position in as few one‐by‐one moves as possible. The test consisted of 21 trials with 3, 4, and 5 colored beads placed on respective number of pegs, with seven problems per puzzle‐type. Derived performance measures were available for 1,860 trauma‐exposed individuals and included average trial time (ATRTI) and excess moves made (EM) for each puzzle‐type separately and once across all puzzle‐types. For the purpose of this study, TOLT performance measures across all puzzle types were used. Consistent with all other measures in this study, data from the last TOLT evaluation were used.

### 2.4 Statistical methods

**Figure 1:**
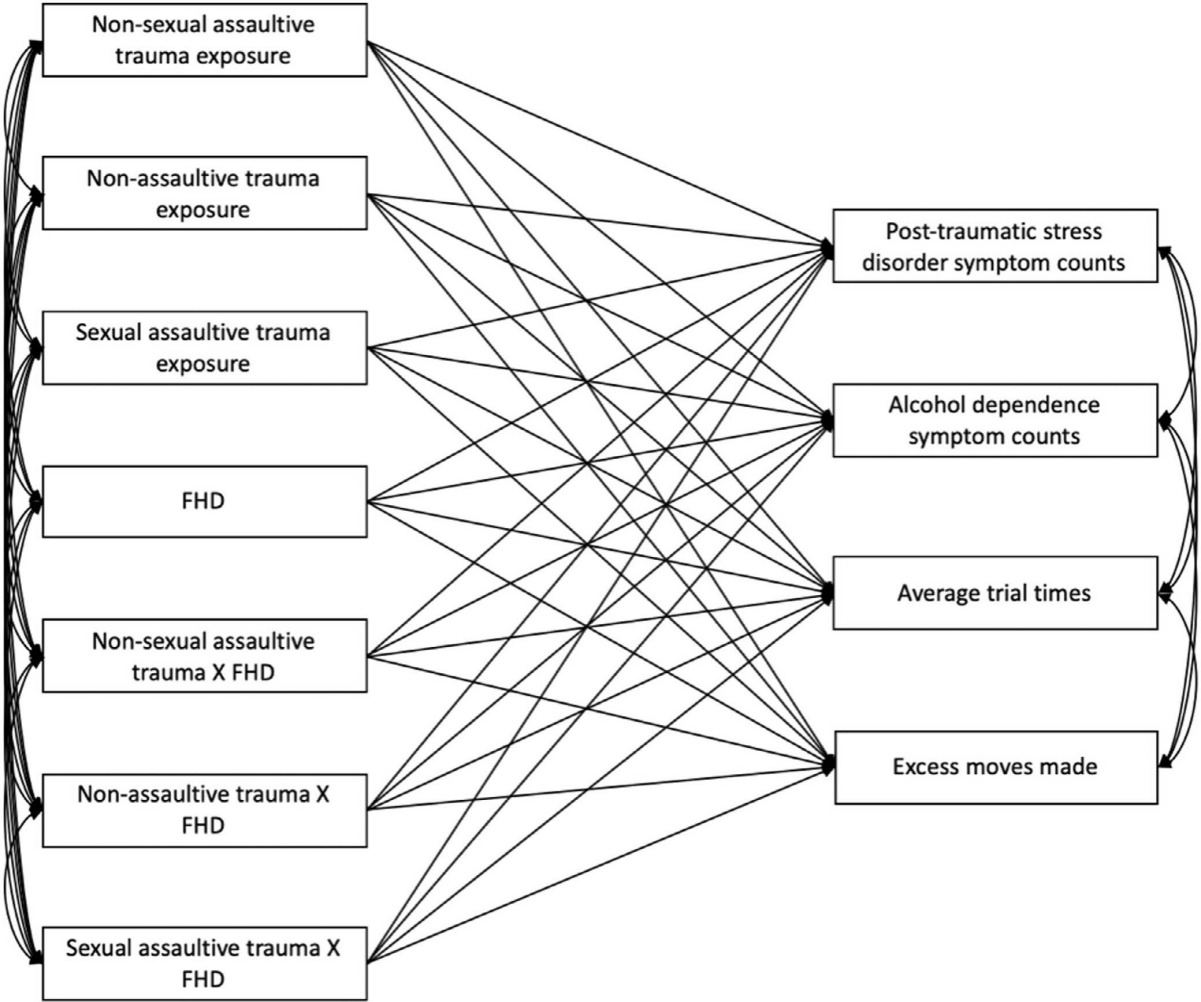
Pathway model used to investigate main and interaction effects of trauma exposure and family history density of alcohol use disorder (FHD) with post‐traumatic stress disorder and alcohol dependence symptom counts, and planning and problem‐solving measures from the Tower of London Test (average trial times, excess moves made). *Note:* Not pictured but are also included in this model are covariates: sex, self‐reported race/ethnicity, age, income, educational attainment (i.e., highest grade completed), number of TOLT sessions.

A primary path analysis was performed using Mplus (Muthén & Muthén, 1998–2017) to investigate main and interaction effects of FHD and three types of trauma (nonsexual assaultive, nonassaultive and sexual assaultive trauma) for DSM‐IV PTSD symptom counts, alcohol dependence symptom counts, and TOLT performance measures (average trial time and excess moves made. Since it is possible that an individual may have multiple types of trauma exposure, all trauma variables were modeled simultaneously. All dependent variables were also modeled simultaneously to account for possible shared liability among PTSD and AD symptom counts and TOLT performance measures (Figure 1). All statistical models were adjusted for familial clustering, sex, self‐reported race/ethnicity, age, income, and educational attainment (i.e., highest grade completed), as each of these variables has been previously shown to influence risk for PTSD and alcohol dependence (Powers, Etkin, Gyurak, Bradley, & Jovanovic, 2015; Tripp, McDevitt‐Murphy, Avery, & Bracken, 2015). Since subjects improve in behavioral tasks over time, and not all individuals have participated in the same number of sessions, number of sessions for each individual was used as a covariate on the TOLT performance measure pathways. Secondary analyses examined the same path model described above stratified by sex, to investigate potential sex differences in all associations.

## 3 RESULTS

Rates of traumatic exposures in COGA’s prospective study have been previously described (Bucholz et al., 2017; McCutcheon et al., 2017; Meyers et al., 2019). Briefly, exposure to nonassaultive trauma was the most prevalent (*N* = 1,479; 79.5%) followed by nonsexual assaultive trauma (*N* = 916; 49.2%) and sexual assaultive trauma (*N* = 364; 19.6%). Rates of sexual trauma were greater among female participants (*N* = 306 vs. *N* = 58), and rates of nonsexual assaultive trauma were greater among male participants (*N* = 616 vs. *N* = 300). 36.7% of individuals who reported experiencing at least one of the three trauma types endorsed at least one PTSD symptom, and 56.1% endorsed at least one DSM‐IV alcohol dependence. See Table 1 for descriptive statistics.

**Table 1:** Demographic information, trauma history, mean DSM‐IV post‐traumatic stress disorder (PTSD) and alcohol dependence (ALC) symptom counts, and mean family history density ratio of alcohol use disorder (AUD) among trauma‐exposed individuals in COGA's prospective study

	Trauma exposed (*n* = 1,860)	Nonsexual assaultive trauma (*n* = 916)	Nonassaultive trauma (*n* = 1,479)	Sexual assaultive trauma (*n* = 364)
Sex				
Female	876 (47.1%)	300 (32.8%)	665 (44.3%)	306 (84.1%)
Male	984 (52.9%)	616 (67.2%)	824 (55.7%)	58 (15.9%)
Self‐Reported Race				
White	993 (53.4%)	444 (48.5%)	781 (52.8%)	212 (58.2%)
Black	615 (33.1%)	346 (37.8%)	500 (33.8%)	104 (28.6%)
Other	252 (13.5%)	126 (13.8%)	198 (13.4%)	48 (13.2%)
Age at most recent interview ‐ mean (*SD*)	21.9 (4.3)	22.4 (4.2)	21.7 (4.2)	23.2 (4.2)
Family history density ratio of AUD‐ mean (*SD*)	0.41 (0.19)	0.42 (0.19)	0.40 (0.19)	0.44 (0.18)
Trauma type				
Nonsexual assaultive	916 (49.2%)	—	652 (44.1%)	152 (41.8%)
Nonassaultive	1,479 (79.5%)	652 (71.2%)	—	209 (57.4%)
Sexual assaultive	364 (19.6%)	152 (16.6%)	209 (14.1%)	—
DSM‐IV symptom counts (*SD*)				
PTSD	3.12 (4.80)	3.68 (5.11)	3.16 (4.84)	6.47 (5.86)
ALC	1.32 (1.66)	1.62 (1.78)	1.32 (1.66)	1.71 (1.95)

**Figure 2:**
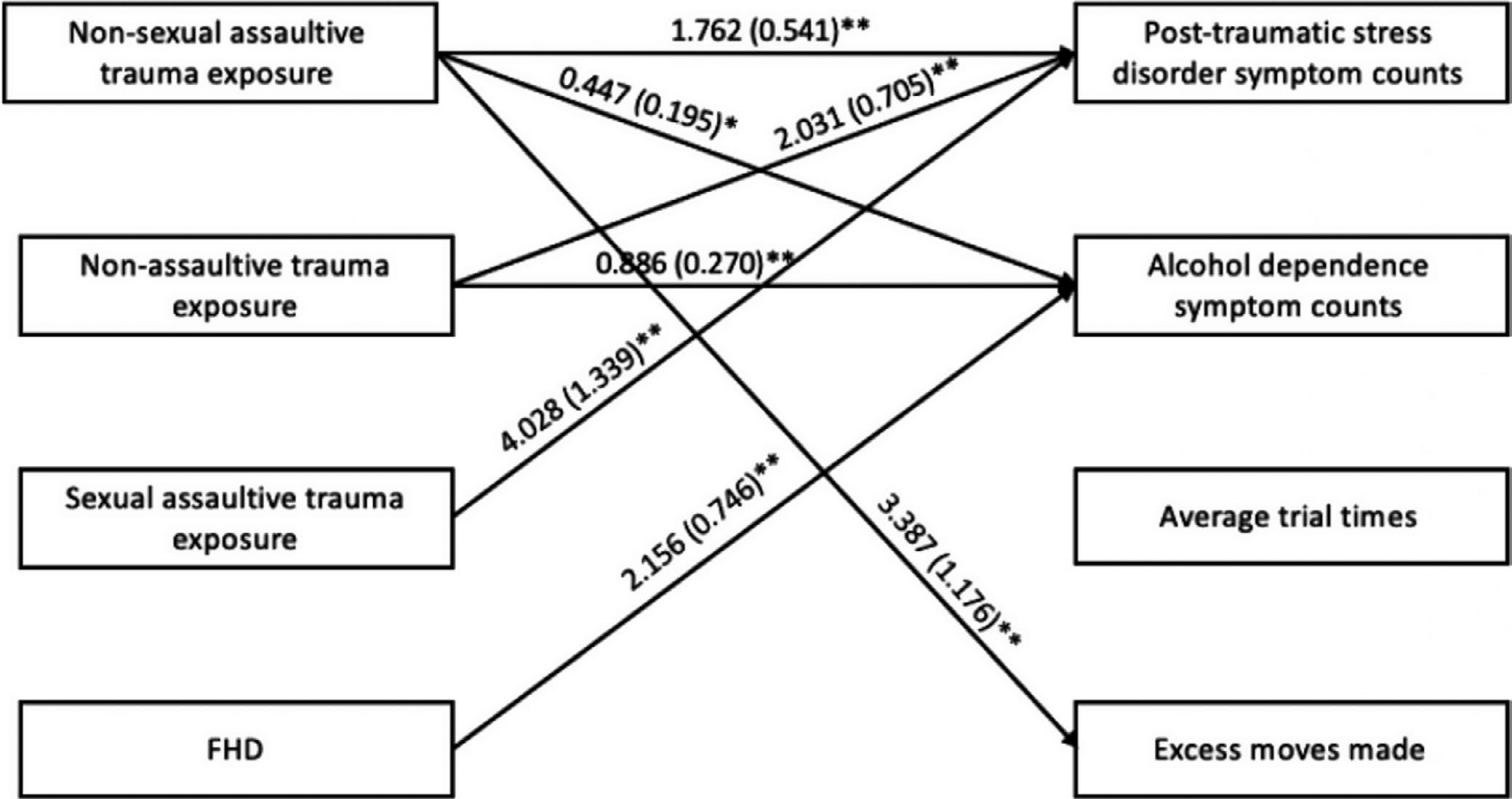
Associations of trauma and family history density of alcohol use disorder (FHD) with post‐traumatic stress disorder symptom counts, alcohol dependence symptom counts, TOLT average trial times, and TOLT excess moves made. *Note:* Only significant pathways are displayed. Not pictured but are also included in this model are interaction variables (non‐sexual assaultive trauma X FHD, non‐assaultive trauma X FHD, sexual assaultive trauma X FHD) and covariates (self‐reported gender, race/ethnicity, age, income, educational attainment (i.e., highest grade completed), number of TOLT sessions). TOLT, Tower of London Test

Consistent with prior studies, significant main effects of nonsexual assaultive, nonassaultive, and sexual assaultive trauma were observed for DSM‐IV PTSD symptom counts such that having any of the three types of trauma was positively associated with PTSD symptom counts (Table 2; Figure 2). Significant main effects of nonsexual assaultive and nonassaultive trauma were observed for DSM‐IV alcohol dependence symptom counts such that having either trauma type was positively associated with alcohol dependence symptom counts (Table 2; Figure 2). A significant main effect of FHD was observed for alcohol dependence symptom counts as well such that as FHD increases, the individual is more likely to have more alcohol dependence symptom counts (Table 2; Figure 2). No moderating effects of FHD on the association between trauma and PTSD or alcohol dependence symptom counts were observed in the full sample.

**Table 2:** Main and interaction effects of trauma exposure, FHD and DSM‐IV PTSD and alcohol dependence (ALC) symptom counts, and TOLT performance measures (average trial time [ATRTI]; excess moves made [EM])

Independent variables^a^	Estimated regression coefficients (standard error)			
	PTSD	ALC	ATRTI	EM
Nonsexual assaultive trauma	2.340 (0.443)^***^	0.338 (0.157)^*^	0.891 (0.576)	3.387 (1.176)^**^
Nonassaultive trauma	2.377 (0.581)^***^	0.474 (0.193)^*^	0.448 (0.733)	−0.559 (1.443)
Sexual assaultive trauma	3.189 (0.741)^***^	0.221 (0.196)	0.398 (0.681)	0.155 (1.433)
Family history density ratio of AUD (FHD)	1.251 (1.403)	1.668 (0.540)^**^	−0.055 (1.638)	−3.530 (3.297)
Nonsexual assaultive trauma*FHD	−1.436 (0.147)	0.386 (0.376)	−0.831 (1.231)	−3.781 (2.508)
Nonassaultive trauma*FHD	−1.157 (0.374)	−0.171 (0.482)	−1.021 (1.594)	2.209 (3.120)
Sexual assaultive trauma*FHD	2.588 (1.556)	0.693 (0.505)	−0.614 (1.391)	2.453 (1.491)

^a^ Sex, race/ethnicity, age, income, and educational attainment reported at last interview were included as covariates, as well as number of interviews. All variables were modeled simultaneously.

* *p* < .05;

** *p* < .01;

*** *p* < .001.

**Figure 3:**
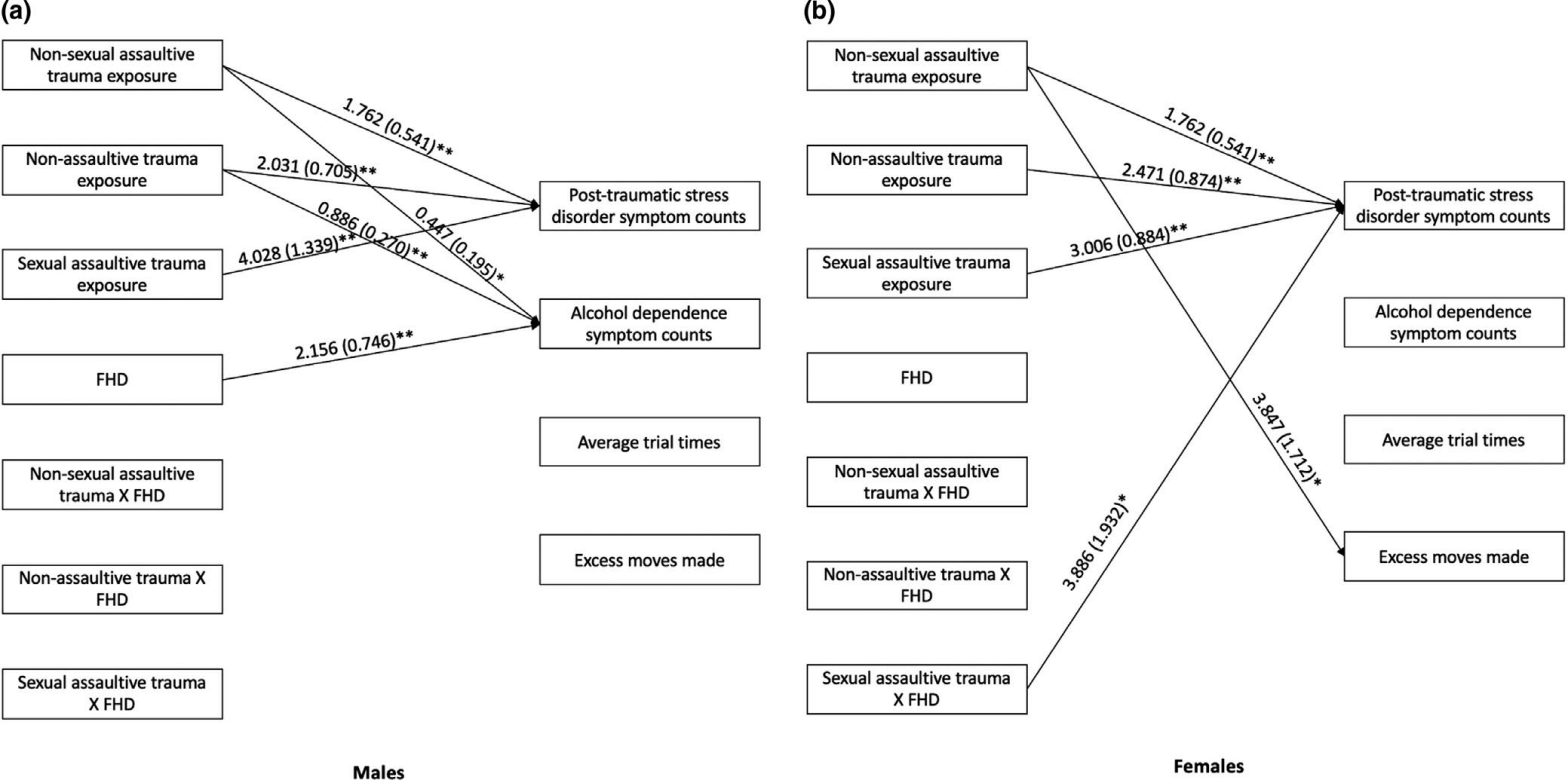
Associations of trauma, family history density of alcohol use disorder (FHD) and trauma‐FHD interactions with post‐traumatic stress disorder symptom counts, alcohol dependence symptom counts, TOLT average trial times, and TOLT excess moves made in male (a) and female (b) participants. *Note:* Only significant pathways are displayed. Not pictured but are also included in this model are covariates: self‐reported race/ethnicity, age, income, educational attainment (i.e., highest grade completed), number of TOLT sessions. TOLT, Tower of London Test

In the sex‐stratified analysis, significant associations were observed such that all three types of trauma were positively associated with PTSD symptom counts in both male and female participants (Table 3; Figure 3). The significant associations of trauma type and FHD observed in the full sample were only observed in male participants, such that male participants, but not female participants, who reported a nonsexual assaultive and nonassaultive trauma, as well as increasing FHD, had more alcohol dependence symptom counts (Table 3; Figure 3a). A significant moderating effect of FHD on the association between sexual assaultive trauma and PTSD symptom counts was observed in female participants, but not male participants, such that sexual assaultive trauma was positively associated with PTSD symptom counts as FHD increases in female participants only (Table 3; Figure 3b).

**Table 3:** Sex differences in main and interaction effects of trauma exposure, FHD and DSM‐IV PTSD and alcohol dependence (ALC) symptom counts, and TOLT performance measures (average trial time [ATRTI]; excess moves made [EM])

Independent variables^*^, ^a^	Estimated regression coefficients (standard error)			
	PTSD	ALC	ATRTI	EM
Male participants				
Nonsexual assaultive trauma	1.762 (0.541)^**^	0.447 (0.195)^*^	0.960 (0.935)	2.957 (1.785)
Nonassaultive trauma	2.031 (0.705)^**^	0.886 (0.270)^**^	−0.289 (1.291)	−1.075 (2.201)
Sexual assaultive trauma	4.028 (1.339)^**^	−0.192 (0.369)	−1.233 (1.893)	−2.220 (2.381)
Family history density of AUD (FHD)	0.807 (1.284)	2.156 (0.746)^**^	−0.381 (2.944)	−3.126 (5.153)
Nonsexual assaultive trauma*FHD	−0.898 (1.284)	0.495 (0.485)	−1.533 (2.008)	−3.365 (3.861)
Nonassaultive trauma*FHD	−0.501 (1.656)	−0.690 (0.680)	0.073 (2.657)	0.987 (4.580)
Sexual assaultive trauma*FHD	−1.399 (2.825)	1.159 (0.873)	1.827 (3.734)	2.514 (4.657)
Female participants				
Nonsexual assaultive trauma	2.948 (0.764)^***^	0.358 (0.232)	1.068 (0.833)	3.847 (1.712)^*^
Nonassaultive trauma	2.471 (0.874)^**^	0.143 (0.265)	1.284 (0.978)	−0.072 (1.883)
Sexual assaultive trauma	3.006 (0.884)^**^	0.158 (0.531)	0.896 (0.806)	1.005 (1.827)
Family history density of AUD (FHD)	0.595 (2.075)	1.156 (0.741)	0.754 (2.140)	−4.086 (4.342)
Nonsexual assaultive trauma*FHD	−1.992 (1.653)	−0.045 (0.550)	−0.593 (1.794)	−4.187 (3.722)
Nonassaultive trauma*FHD	−1.028 (2.009)	0.323 (0.690)	−2.349 (2.122)	3.471 (4.154)
Sexual assaultive trauma*FHD	3.886 (1.932)^*^	1.022 (0.631)	−1.276 (1.675)	2.580 (3.913)

^a^ Race/ethnicity, age, income, and educational attainment reported at last interview were included as covariates, as well as number of interviews. All variables were modeled simultaneously. Bolded results indicate significance in one sex, but not the other.

* *p* < .05;

** *p* < .01;

*** *p* < .001.

A main significant effect was observed for nonsexual assaultive trauma and the TOLT excess moves made, such that having a nonsexual assaultive trauma was positively associated with excess moves made (i.e., more excess moves made; Table 2, Figure 2). In the sex‐stratified analysis, this association was only observed in female participants (Table 3, Figure 3b). No significant associations were observed for average trial time in the full or sex‐stratified samples. No moderating effects of FHD on the associations between trauma and TOLT performance measures were observed in either the full or sex‐stratified analyses.

## 4 DISCUSSION

Consistent with prior research, trauma exposure was positively associated with PTSD and alcohol dependence symptom counts, and FHD was positively associated with alcohol dependence symptom counts. While FHD did not moderate the associations between trauma and PTSD in the full sample, a sex‐stratified analysis showed that the association between sexual assaultive trauma and PTSD symptom counts was moderated by FHD in female participants only. One trauma type, nonsexual assaultive trauma, was associated with more TOLT excess moves made, which was shown in the sex‐stratified analysis to be driven by the female participants.

### 4.1 Sex differences in the moderating effects on the association of trauma and DSM‐IV PTSD symptom counts

This study extends previous work demonstrating that trauma is associated with risk for PTSD and alcohol dependence, as well as associations between FHD and the risk for alcohol dependence (Breslau et al., 2013; Giaconia et al., 1995; Knopik et al., 2004; Pandey et al., 2020), by examining these important risk factors together with measures of executive functioning in an integrated model. Findings from this study demonstrate that the association between trauma, specifically sexual assaultive trauma, and PTSD is moderated by FHD, such that increasing FHD increased the association with PTSD in female participants with sexual assaultive trauma, but not nonsexual assaultive or nonassaultive trauma. While nonsexual assaultive and nonassaultive traumatic exposures were both positively associated with PTSD symptom counts in male and female participants separately as well as in the combined sample, the effect sizes were smaller than sexual assaultive trauma. This may suggest that although nonsexual assaultive and nonassaultive traumatic exposures have an effect on future symptoms of PTSD, sexual assaultive trauma may affect women to a greater extent than other forms of trauma. Many studies typically combine nonsexual assaultive trauma and sexual assaultive trauma into a single trauma category (assaultive trauma). Together with previous work (Meyers et al., 2019), the results in this study suggest that these traumatic exposures may affect male and female individuals differently and should be considered as separate traumatic exposures in future studies, in addition to considering sex differences.

Since research has shown that sexual assault is most commonly associated with PTSD in women (Kessler et al., 1995), it is possible that other risk factors for PTSD development, such as a FH of alcohol dependence, may increase the risk for developing PTSD to a greater extent or exacerbate the effects of exposure to sexual assaultive trauma in women, which could explain our observed result that increasing FHD of alcohol dependence increased the association with PTSD in female participants with sexual assaultive trauma. Prior studies have shown that children who grow up in a household with at least one parent with alcohol dependence are more likely to experience trauma compared to children with unaffected parents (Dube et al., 2001; Meyers et al., 2014). However, these studies have only examined dichotomous parental alcohol dependence status, whereas FHD measures may be more useful (Cservenka et al., 2015; Pandey et al., 2020; Powers et al., 2017). Future studies should investigate how different levels of FHD may influence PTSD symptoms and risk, as well as examine molecular genetic risk of alcohol dependence on PTSD in female individuals who have been exposed to a sexual assaultive trauma.

### 4.2 Association of trauma and FHD on planning and problem‐solving aspects of executive function

Overall, our findings suggest that nonsexual assaultive trauma was associated with excess moves made, which may suggest poor planning and problem‐solving skills. Although previous studies have shown associations between early trauma and poor cognitive function, this is the first study investigating the influence of different types of trauma in a planning and problem‐solving task, such as the TOLT (Bucker et al., 2012; Corbo, Amick, Milberg, McGlinchey, & Salat, 2016; DePrince, Weinzierl, & Combs, 2009; Malarbi, Abu‐Rayya, Muscara, & Stargatt, 2017). For example, Corbo et al. (2016) showed that veterans with a history of childhood sexual abuse, physical abuse, or family violence had a greater number of errors in an affective Go/No‐Go task. DePrince et al. (2009) showed that familial trauma (relative to nonfamilial and no trauma exposure) was associated with poor performance, using an executive function composite score. Further, Malarbi et al. (2017) demonstrated that trauma‐exposed children showed cognitive deficits, especially those with a PTSD diagnosis. However, these studies have been limited by sample sizes (e.g., Bucker et al. (2012) recruited 30 trauma‐exposed children and 30 age‐ and sex‐matched controls) or available data on trauma type (Corbo et al., 2016; Malarbi et al., 2017). One study has shown that sexual trauma, and not physical or interpersonal trauma, moderates psychotherapy outcome for PTSD (Markowitz, Neria, Lovell, Van Meter, & Petkova, 2017), which underscores the importance of investigating trauma by type in order to better understand the development of PTSD and related disorders. While these results indicate that there are associations between trauma and poor problem‐solving (i.e., more excess moves made), this result is based on only a single test (TOLT).

Given the heterogeneous nature of PTSD and the results of our study, perhaps one specific pathway to developing PTSD symptoms could be investigated by linking trauma type, specifically nonsexual assaultive trauma, to cognitive function. Individuals who have been exposed to nonsexual assaultive trauma are more likely to develop PTSD compared to those exposed to nonassaultive trauma (Cisler et al., 2011; Kessler et al., 2005; Ozer et al., 2003; Resnick et al., 1993), and poor executive functioning has been observed among individuals with PTSD (Flaks et al., 2014; Olff et al., 2014). Therefore, it is possible that the poor executive functioning observed in individuals with PTSD may be influenced by a specific trauma type, which the current study suggests may be nonsexual assaultive trauma. This suggests that physical traumatic exposure, such as nonsexual assaultive trauma, when experienced during the critical developmental period of adolescence, may influence the development of the prefrontal cortex and lead to poor problem‐solving skills. While this association was observed in the full sample, in the sex‐stratified analysis, this association was only significant in female participants who have experienced a nonsexual assaultive trauma, suggesting that sexual assaultive trauma may influence planning/problem‐solving ability in female individuals. While studies have investigated sex differences in planning and problem‐solving tasks, such as the TOLT, no study to our knowledge has investigated the influences of trauma type on planning and problem‐solving ability of executive function in male and female individuals separately. Future studies should investigate sex differences and trauma type when considering the effects of trauma on planning and problem‐solving aspects of executive function and should also investigate whether early trauma directly influences the development of executive functions, as well as PTSD and alcohol dependence in adulthood.

### 4.3 Limitations

The presented study utilized data from the final assessment from COGA’s prospective study, and therefore inferences regarding causal relationships between trauma, planning/problem‐solving aspects of executive functioning, and DSM‐IV PTSD and alcohol dependence diagnoses cannot be determined. Further, since the data used were across four potential interviews, it is possible that PTSD and alcohol dependence symptoms appeared several years apart from each other or may have not appeared yet in the individual. Future work should expand on these findings by examining temporality of PTSD and alcohol dependence in a longitudinal framework. While a nonresponse analysis indicated that individuals who did not return for follow‐up in this study showed no differences regarding sex, race/ethnicity, sexual trauma exposure, or TOLT measures, attrition may have impacted this study's findings. Therefore, this study only included participants with data from baseline to follow‐up 3 to allow for the greatest proportion of nonmissing data for the analyses. In addition, the SSAGA interview only asks the participants about the first traumatic exposure experience for each traumatic exposure. Therefore, this study did not account for recurrent exposure to trauma.

## 5 CONCLUSIONS

Our study has shown that increasing FHD of alcohol dependence in sexual assaultive trauma‐exposed female participants was associated with increased PTSD symptom counts. Nonsexual assaultive trauma was associated with poorer TOLT task performance in our full sample. In the sex‐stratified analysis, this association was only observed in female participants. These findings suggest that specific types of traumatic exposure are more likely to be associated with PTSD and planning and problem‐solving aspects of executive function. Future studies should explore whether any observed poor planning/problem‐solving performance precedes or follows trauma exposure, considering trauma type and sex differences. Further, future studies should investigate whether these aspects of executive function precede or follow PTSD and/or alcohol dependence onset. Understanding the complex etiology of these commonly comorbid disorders may inform early intervention and treatment strategies aimed at reducing the severity and endurance of PTSD and alcohol dependence.

## CONFLICT OF INTEREST

There are no conflicts of interest to disclose.

## **AUTHOR** **CONTRIBUTION**

Stacey Subbie‐Saenz de Viteri involved in manuscript preparation, conception and design of analyses, analysis, and interpretation of data. Ashwini Pandey involved in manuscript revision, conception and design of analyses, and interpretation of data. Gayathri Pandey involved in manuscript revision and interpretation of data. Chella Kamarajan involved in manuscript revision and interpretation of data. Rebecca Smith involved in manuscript preparation and revision. Andrey Anokhin, Lance Bauer, Annah Bender, Grace Chan, Danielle Dick, Howard Edenberg, Sivan Kinreich, John Kramer, Marc Schuckit, Marc Schuckit, and Yong Zang involved in manuscript revision. Vivia McCutcheon involved in manuscript revision, interpretation of data. Kathleen Bucholz and Bernice Porjesz involved in manuscript revision, conception and design of data acquisition, and interpretation of data. Jacquelyn L. Meyers involved in manuscript preparation and revision, conception and design of analyses, and interpretation of data.

### Peer Review

The peer review history for this article is available at https://publons.com/publon/10.1002/brb3.1789.

## Data Availability

The data that support the findings of this study are available from the corresponding author upon reasonable request.

## References

[brb31789-bib-0001] Adkison, S. E. , Grohman, K. , Colder, C. R. , Leonard, K. , Orrange‐Torchia, T. , Peterson, E. , & Eiden, R. D. (2013). Impact of fathers' alcohol problems on the development of effortful control in early adolescence. Journal of Studies on Alcohol and Drugs, 74(5), 674–683. 10.15288/jsad.2013.74.674 23948526 PMC3749310

[brb31789-bib-0002] Allen, J. P. , Crawford, E. F. , & Kudler, H. (2016). Nature and treatment of comorbid alcohol problems and post traumatic stress disorder among American Military Personnel and Veterans. Alcohol Research, 38(1), 133–140. 27159820 10.35946/arcr.v38.1.16 PMC4872608

[brb31789-bib-0003] American Psychiatric Association (2000). Diagnostic criteria from DSM‐IV‐TR. Washington, DC: American Psychiatric Association.

[brb31789-bib-0004] Aupperle, R. L. , Melrose, A. J. , Stein, M. B. , & Paulus, M. P. (2012). Executive function and PTSD: Disengaging from trauma. Neuropharmacology, 62(2), 686–694. 10.1016/j.neuropharm.2011.02.008 21349277 PMC4719148

[brb31789-bib-0005] Baker, S. C. , Rogers, R. D. , Owen, A. M. , Frith, C. D. , Dolan, R. J. , Frackowiak, R. S. , & Robbins, T. W. (1996). Neural systems engaged by planning: A PET study of the Tower of London task. Neuropsychologia, 34(6), 515–526. 10.1016/0028-3932(95)00133-6 8736565

[brb31789-bib-0006] Banerjee, S. B. , Morrison, F. G. , & Ressler, K. J. (2017). Genetic approaches for the study of PTSD: Advances and challenges. Neuroscience Letters, 649, 139–146. 10.1016/j.neulet.2017.02.058 28242325 PMC5470933

[brb31789-bib-0007] Beals, J. , Belcourt‐Dittloff, A. , Garroutte, E. M. , Croy, C. , Jervis, L. L. , Whitesell, N. R. , … Manson, S. M. (2013). Trauma and conditional risk of posttraumatic stress disorder in two American Indian reservation communities. Social Psychiatry and Psychiatric Epidemiology, 48(6), 895–905. 10.1007/s00127-012-0615-5 23135256 PMC3578964

[brb31789-bib-0008] Bender, A. K. K. , Meyers, J. , McCutcheon, V. V. , & Bucholz, K. (2016). Trauma exposure and PTSD among young adult offspring of parents with alcohol problems. Paper presented at the Research Society on Alcoholism, New Orleans, LA.

[brb31789-bib-0009] Blanco, C. , Xu, Y. , Brady, K. , Perez‐Fuentes, G. , Okuda, M. , & Wang, S. (2013). Comorbidity of posttraumatic stress disorder with alcohol dependence among US adults: Results from National Epidemiological Survey on Alcohol and Related Conditions. Drug and Alcohol Dependence, 132(3), 630–638. 10.1016/j.drugalcdep.2013.04.016 23702490 PMC3770804

[brb31789-bib-0010] Boudoukha, A. H. , Ouagazzal, O. , & Goutaudier, N. (2017). When traumatic event exposure characteristics matter: Impact of traumatic event exposure characteristics on posttraumatic and dissociative symptoms. Psychological Trauma, 9(5), 561–566. 10.1037/tra0000243 27929307

[brb31789-bib-0011] Breslau, N. (2009). The epidemiology of trauma, PTSD, and other posttrauma disorders. Trauma Violence Abuse, 10(3), 198–210. 10.1177/1524838009334448 19406860

[brb31789-bib-0012] Breslau, N. , Kessler, R. C. , Chilcoat, H. D. , Schultz, L. R. , Davis, G. C. , & Andreski, P. (1998). Trauma and posttraumatic stress disorder in the community: The 1996 Detroit Area Survey of Trauma. Archives of General Psychiatry, 55(7), 626–632. 10.1001/archpsyc.55.7.626 9672053

[brb31789-bib-0013] Breslau, N. , Troost, J. P. , Bohnert, K. , & Luo, Z. (2013). Influence of predispositions on post‐traumatic stress disorder: Does it vary by trauma severity? Psychological Medicine, 43(2), 381–390. 10.1017/S0033291712001195 22703614 PMC3924550

[brb31789-bib-0014] Bucholz, K. K. , Cadoret, R. , Cloninger, C. R. , Dinwiddie, S. H. , Hesselbrock, V. M. , Nurnberger, J. I. , … Schuckit, M. A. (1994). A new, semi‐structured psychiatric interview for use in genetic linkage studies: A report on the reliability of the SSAGA. Journal of Studies on Alcohol, 55(2), 149–158. 10.15288/jsa.1994.55.149 8189735

[brb31789-bib-0015] Bucholz, K. K. , McCutcheon, V. V. , Agrawal, A. , Dick, D. M. , Hesselbrock, V. M. , Kramer, J. R. , … Porjesz, B. (2017). Comparison of parent, peer, psychiatric, and cannabis use influences across stages of offspring alcohol involvement: Evidence from the COGA prospective study. Alcoholism, Clinical and Experimental Research, 41(2), 359–368. 10.1111/acer.13293 PMC5272776 28073157

[brb31789-bib-0016] Bücker, J. , Kapczinski, F. , Post, R. , Ceresér, K. M. , Szobot, C. , Yatham, L. N. , … Kauer‐Sant'Anna, M. (2012). Cognitive impairment in school‐aged children with early trauma. Comprehensive Psychiatry, 53(6), 758–764. 10.1016/j.comppsych.2011.12.006 22300905

[brb31789-bib-0017] Chung, H. , & Breslau, N. (2008). The latent structure of post‐traumatic stress disorder: Tests of invariance by gender and trauma type. Psychological Medicine, 38(4), 563–573. 10.1017/S0033291707002589 18325132

[brb31789-bib-0018] Cisler, J. M. , Amstadter, A. B. , Begle, A. M. , Resnick, H. S. , Danielson, C. K. , Saunders, B. E. , & Kilpatrick, D. G. (2011). PTSD symptoms, potentially traumatic event exposure, and binge drinking: A prospective study with a national sample of adolescents. Journal of Anxiety Disorders, 25(7), 978–987. 10.1016/j.janxdis.2011.06.006 21783340 PMC3546501

[brb31789-bib-0019] Corbo, V. , Amick, M. A. , Milberg, W. P. , McGlinchey, R. E. , & Salat, D. H. (2016). Early life trauma is associated with altered white matter integrity and affective control. Journal of Psychiatric Research, 79, 70–77. 10.1016/j.jpsychires.2016.05.001 27214523

[brb31789-bib-0020] Cservenka, A. , Gillespie, A. J. , Michael, P. G. , & Nagel, B. J. (2015). Family history density of alcoholism relates to left nucleus accumbens volume in adolescent girls. Journal of Studies on Alcohol and Drugs, 76(1), 47–56. 25486393 PMC4263780

[brb31789-bib-0021] Davis, H. P. K. (2002). Colorado Assessment Tests (CATs) (Version 1.2). Colorado Springs, Colorado.

[brb31789-bib-0022] DePrince, A. P. , Weinzierl, K. M. , & Combs, M. D. (2009). Executive function performance and trauma exposure in a community sample of children. Child Abuse and Neglect, 33(6), 353–361. 10.1016/j.chiabu.2008.08.002 19477515

[brb31789-bib-0023] Dube, S. R. , Anda, R. F. , Felitti, V. J. , Croft, J. B. , Edwards, V. J. , & Giles, W. H. (2001). Growing up with parental alcohol abuse: Exposure to childhood abuse, neglect, and household dysfunction. Child Abuse and Neglect, 25(12), 1627–1640. 10.1016/s0145-2134(01)00293-9 11814159

[brb31789-bib-0024] Dube, S. R. , Felitti, V. J. , Dong, M. , Chapman, D. P. , Giles, W. H. , & Anda, R. F. (2003). Childhood abuse, neglect, and household dysfunction and the risk of illicit drug use: The adverse childhood experiences study. Pediatrics, 111(3), 564–572. 10.1542/peds.111.3.564 12612237

[brb31789-bib-0025] Edwards, A. C. , Heron, J. , Vladimirov, V. , Wolen, A. R. , Adkins, D. E. , Aliev, F. , … Kendler, K. S. (2017). The rate of change in alcohol misuse across adolescence is heritable. Alcoholism, Clinical and Experimental Research, 41(1), 57–64. 10.1111/acer.13262 PMC5205550 27892595

[brb31789-bib-0026] Edwards, A. C. , & Kendler, K. S. (2013). Alcohol consumption in men is influenced by qualitatively different genetic factors in adolescence and adulthood. Psychological Medicine, 43(9), 1857–1868. 10.1017/S0033291712002917 23282961 PMC3670965

[brb31789-bib-0027] Erol, A. , & Karpyak, V. M. (2015). Sex and gender‐related differences in alcohol use and its consequences: Contemporary knowledge and future research considerations. Drug and Alcohol Dependence, 156, 1–13. 10.1016/j.drugalcdep.2015.08.023 26371405

[brb31789-bib-0028] Flaks, M. K. , Malta, S. M. , Almeida, P. P. , Bueno, O. F. A. , Pupo, M. C. , Andreoli, S. B. , … Bressan, R. A. (2014). Attentional and executive functions are differentially affected by post‐traumatic stress disorder and trauma. Journal of Psychiatric Research, 48(1), 32–39. 10.1016/j.jpsychires.2013.10.009 24199652

[brb31789-bib-0029] Follette, V. M. , Polusny, M. A. , Bechtle, A. E. , & Naugle, A. E. (1996). Cumulative trauma: The impact of child sexual abuse, adult sexual assault, and spouse abuse. Journal of Traumatic Stress, 9(1), 25–35. 10.1007/bf02116831 8750449

[brb31789-bib-0030] Fowler, T. , Shelton, K. , Lifford, K. , Rice, F. , McBride, A. , Nikolov, I. , … van den Bree, M. B. M. (2007). Genetic and environmental influences on the relationship between peer alcohol use and own alcohol use in adolescents. Addiction, 102(6), 894–903. 10.1111/j.1360-0443.2007.01824.x 17523983 PMC1974773

[brb31789-bib-0031] Giaconia, R. M. , Reinherz, H. Z. , Silverman, A. B. , Pakiz, B. , Frost, A. K. , & Cohen, E. (1995). Traumas and posttraumatic stress disorder in a community population of older adolescents. Journal of the American Academy of Child and Adolescent Psychiatry, 34(10), 1369–1380. 10.1097/00004583-199510000-00023 7592275

[brb31789-bib-0032] Goldstein, B. , Bradley, B. , Ressler, K. J. , & Powers, A. (2017). Associations between posttraumatic stress disorder, emotion dysregulation, and alcohol dependence symptoms among inner city females. Journal of Clinical Psychology, 73(3), 319–330. 10.1002/jclp.22332 27467499 PMC5324595

[brb31789-bib-0033] Green, B. L. , Goodman, L. A. , Krupnick, J. L. , Corcoran, C. B. , Petty, R. M. , Stockton, P. , & Stern, N. M. (2000). Outcomes of single versus multiple trauma exposure in a screening sample. Journal of Traumatic Stress, 13(2), 271–286. 10.1023/A:1007758711939 10838675

[brb31789-bib-0034] Hasin, D. S. , Stinson, F. S. , Ogburn, E. , & Grant, B. F. (2007). Prevalence, correlates, disability, and comorbidity of DSM‐IV alcohol abuse and dependence in the United States: Results from the National Epidemiologic Survey on Alcohol and Related Conditions. Archives of General Psychiatry, 64(7), 830–842. 10.1001/archpsyc.64.7.830 17606817

[brb31789-bib-0035] Hien, D. , Cohen, L. , & Campbell, A. (2005). Is traumatic stress a vulnerability factor for women with substance use disorders? Clin Psychol Rev, 25(6), 813–823. 10.1016/j.cpr.2005.05.006 15967556 PMC3679552

[brb31789-bib-0036] Hussey, J. M. , Chang, J. J. , & Kotch, J. B. (2006). Child maltreatment in the United States: Prevalence, risk factors, and adolescent health consequences. Pediatrics, 118(3), 933–942. 10.1542/peds.2005-2452 16950983

[brb31789-bib-0037] Juraska, J. M. , & Willing, J. (2017). Pubertal onset as a critical transition for neural development and cognition. Brain Research, 1654(Pt B), 87–94. 10.1016/j.brainres.2016.04.012 27060769 PMC5053848

[brb31789-bib-0038] Kanagaratnam, P. , & Asbjornsen, A. E. (2007). Executive deficits in chronic PTSD related to political violence. Journal of Anxiety Disorders, 21(4), 510–525. 10.1016/j.janxdis.2006.06.008 16938424

[brb31789-bib-0039] Kessler, R. C. , Chiu, W. T. , Demler, O. , Merikangas, K. R. , & Walters, E. E. (2005). Prevalence, severity, and comorbidity of 12‐month DSM‐IV disorders in the National Comorbidity Survey Replication. Archives of General Psychiatry, 62(6), 617–627. 10.1001/archpsyc.62.6.617 15939839 PMC2847357

[brb31789-bib-0040] Kessler, R. C. , Crum, R. M. , Warner, L. A. , Nelson, C. B. , Schulenberg, J. , & Anthony, J. C. (1997). Lifetime co‐occurrence of DSM‐III‐R alcohol abuse and dependence with other psychiatric disorders in the National Comorbidity Survey. Archives of General Psychiatry, 54(4), 313–321. 10.1001/archpsyc.1997.01830160031005 9107147

[brb31789-bib-0041] Kessler, R. C. , Sonnega, A. , Bromet, E. , Hughes, M. , & Nelson, C. B. (1995). Posttraumatic stress disorder in the National Comorbidity Survey. Archives of General Psychiatry, 52(12), 1048–1060. 10.1001/archpsyc.1995.03950240066012 7492257

[brb31789-bib-0042] Keyes, K. M. , Hatzenbuehler, M. L. , & Hasin, D. S. (2011). Stressful life experiences, alcohol consumption, and alcohol use disorders: The epidemiologic evidence for four main types of stressors. Psychopharmacology (Berl), 218(1), 1–17. 10.1007/s00213-011-2236-1 21373787 PMC3755727

[brb31789-bib-0043] Khoury, L. , Tang, Y. L. , Bradley, B. , Cubells, J. F. , & Ressler, K. J. (2010). Substance use, childhood traumatic experience, and Posttraumatic Stress Disorder in an urban civilian population. Depress Anxiety, 27(12), 1077–1086. 10.1002/da.20751 21049532 PMC3051362

[brb31789-bib-0044] Kilpatrick, D. G. , Resnick, H. S. , Milanak, M. E. , Miller, M. W. , Keyes, K. M. , & Friedman, M. J. (2013). National estimates of exposure to traumatic events and PTSD prevalence using DSM‐IV and DSM‐5 criteria. Journal of Traumatic Stress, 26(5), 537–547. 10.1002/jts.21848 24151000 PMC4096796

[brb31789-bib-0045] Knopik, V. S. , Heath, A. C. , Madden, P. A. F. , Bucholz, K. K. , Slutske, W. S. , Nelson, E. C. , … Martin, N. G. (2004). Genetic effects on alcohol dependence risk: Re‐evaluating the importance of psychiatric and other heritable risk factors. Psychological Medicine, 34(8), 1519–1530. 10.1017/s0033291704002922 15724882

[brb31789-bib-0046] Koob, G. F. , & Volkow, N. D. (2016). Neurobiology of addiction: A neurocircuitry analysis. Lancet Psychiatry, 3(8), 760–773. 10.1016/S2215-0366(16)00104-8 27475769 PMC6135092

[brb31789-bib-0047] Lagarde, G. , Doyon, J. , & Brunet, A. (2010). Memory and executive dysfunctions associated with acute posttraumatic stress disorder. Psychiatry Research, 177(1–2), 144–149. 10.1016/j.psychres.2009.02.002 20381880

[brb31789-bib-0048] Lang, A. J. , Rodgers, C. S. , Laffaye, C. , Satz, L. E. , Dresselhaus, T. R. , & Stein, M. B. (2003). Sexual trauma, posttraumatic stress disorder, and health behavior. Behavioral Medicine, 28(4), 150–158. 10.1080/08964280309596053 14663922

[brb31789-bib-0049] Laviola, G. , & Marco, E. M. (2011). Passing the knife edge in adolescence: Brain pruning and specification of individual lines of development. Neuroscience and Biobehavioral Reviews, 35(8), 1631–1633. 10.1016/j.neubiorev.2011.05.011 21645542

[brb31789-bib-0050] Logrip, M. L. , Zorrilla, E. P. , & Koob, G. F. (2012). Stress modulation of drug self‐administration: Implications for addiction comorbidity with post‐traumatic stress disorder. Neuropharmacology, 62(2), 552–564. 10.1016/j.neuropharm.2011.07.007 21782834 PMC3206986

[brb31789-bib-0051] Malarbi, S. , Abu‐Rayya, H. M. , Muscara, F. , & Stargatt, R. (2017). Neuropsychological functioning of childhood trauma and post‐traumatic stress disorder: A meta‐analysis. Neuroscience and Biobehavioral Reviews, 72, 68–86. 10.1016/j.neubiorev.2016.11.004 27851897

[brb31789-bib-0052] Markowitz, J. C. , Neria, Y. , Lovell, K. , Van Meter, P. E. , & Petkova, E. (2017). History of sexual trauma moderates psychotherapy outcome for posttraumatic stress disorder. Depress Anxiety, 34(8), 692–700. 10.1002/da.22619 28376282 PMC5542864

[brb31789-bib-0053] McCutcheon, V. V. , Schuckit, M. A. , Kramer, J. R. , Chan, G. , Edenberg, H. J. , Smith, T. L. , … Bucholz, K. K. (2017). Familial association of abstinent remission from alcohol use disorder in first‐degree relatives of alcohol‐dependent treatment‐seeking probands. Addiction, 112(11), 1909–1917. 10.1111/add.13890 28556494 PMC5633502

[brb31789-bib-0054] Meyers, J. , McCutcheon, V. V. , Pandey, A. K. , Kamarajan, C. , Subbie, S. , Chorlian, D. , … Porjesz, B. (2019). Early sexual trauma exposure and neural response inhibition in adolescence and young adults: Trajectories of frontal theta oscillations during a go/no‐go task. Journal of the American Academy of Child and Adolescent Psychiatry, 58(2), 242–255 e242. 10.1016/j.jaac.2018.07.905 30738551 PMC6537865

[brb31789-bib-0055] Meyers, J. L. , Shmulewitz, D. , Elliott, J. C. , Thompson, R. G. , Aharonovich, E. , Spivak, B. , … Hasin, D. S. (2014). Parental alcohol history differentially predicts offspring disorders in distinct subgroups in Israel. J Stud Alcohol Drugs, 75(5), 859–869. 10.15288/jsad.2014.75.859 25208204 PMC4161705

[brb31789-bib-0056] Muthén, L. K. , & Muthén, B. O. (1998–2017). Mplus user’s guide (Vol. 8). Los Angeles, CA: Muthén & Muthén.

[brb31789-bib-0057] Nugent, N. R. , Amstadter, A. B. , & Koenen, K. C. (2008). Genetics of post‐traumatic stress disorder: Informing clinical conceptualizations and promoting future research. American Journal of Medical Genetics Part C: Seminars in Medical Genetics, 148C(2), 127–132. 10.1002/ajmg.c.30169 PMC2680188 18412098

[brb31789-bib-0058] Olff, M. , Polak, A. R. , Witteveen, A. B. , & Denys, D. (2014). Executive function in posttraumatic stress disorder (PTSD) and the influence of comorbid depression. Neurobiology of Learning and Memory, 112, 114–121. 10.1016/j.nlm.2014.01.003 24440596

[brb31789-bib-0059] Ozer, E. J. , Best, S. R. , Lipsey, T. L. , & Weiss, D. S. (2003). Predictors of posttraumatic stress disorder and symptoms in adults: A meta‐analysis. Psychological Bulletin, 129(1), 52–73. 10.1037/0033-2909.129.1.52 12555794

[brb31789-bib-0060] Packwood, S. , Hodgetts, H. M. , & Tremblay, S. (2011). A multiperspective approach to the conceptualization of executive functions. Journal of Clinical and Experimental Neuropsychology, 33(4), 456–470. 10.1080/13803395.2010.533157 21271425

[brb31789-bib-0061] Pandey, A. K. , Ardekani, B. A. , Kamarajan, C. , Zhang, J. , Chorlian, D. B. , Byrne, K.‐H. , … Porjesz, B. (2018). Lower prefrontal and hippocampal volume and diffusion tensor imaging differences reflect structural and functional abnormalities in abstinent individuals with alcohol use disorder. Alcoholism, Clinical and Experimental Research, 42(10), 1883–1896. 10.1111/acer.13854 PMC6167190 30118142

[brb31789-bib-0062] Pandey, G. , Seay, M. J. , Meyers, J. L. , Chorlian, D. B. , Pandey, A. K. , Kamarajan, C. , … Porjesz, B. (2020). Density and dichotomous family history measures of alcohol use disorder as predictors of behavioral and neural phenotypes: A comparative study across gender and race/ethnicity. Alcoholism: Clinical and Experimental Research, 44(3), 697–710. 10.1111/acer.14280. PMC8357185 31957047

[brb31789-bib-0063] Pennington, B. F. , & Ozonoff, S. (1996). Executive functions and developmental psychopathology. Journal of Child Psychology and Psychiatry, 37(1), 51–87. 10.1111/j.1469-7610.1996.tb01380.x 8655658

[brb31789-bib-0064] Powers, A. , Etkin, A. , Gyurak, A. , Bradley, B. , & Jovanovic, T. (2015). Associations Between childhood abuse, posttraumatic stress disorder, and implicit emotion regulation deficits: Evidence from a low‐income, inner‐city population. Psychiatry, 78(3), 251–264. 10.1080/00332747.2015.1069656 26391833 PMC4705548

[brb31789-bib-0065] Powers, G. , Berger, L. , Fuhrmann, D. , & Fendrich, M. (2017). Family history density of substance use problems among undergraduate college students: Associations with heavy alcohol use and alcohol use disorder. Addictive Behaviors, 71, 1–6. 10.1016/j.addbeh.2017.02.015 28231492

[brb31789-bib-0066] Reilly, M. T. , Noronha, A. , Goldman, D. , & Koob, G. F. (2017). Genetic studies of alcohol dependence in the context of the addiction cycle. Neuropharmacology, 122, 3–21. 10.1016/j.neuropharm.2017.01.017 28118990 PMC6233301

[brb31789-bib-0067] Resnick, H. S. , Kilpatrick, D. G. , Dansky, B. S. , Saunders, B. E. , & Best, C. L. (1993). Prevalence of civilian trauma and posttraumatic stress disorder in a representative national sample of women. Journal of Consulting and Clinical Psychology, 61(6), 984–991. 10.1037//0022-006x.61.6.984 8113499

[brb31789-bib-0068] Ruocco, A. C. , Rodrigo, A. H. , Lam, J. , Di Domenico, S. I. , Graves, B. , & Ayaz, H. (2014). A problem‐solving task specialized for functional neuroimaging: Validation of the Scarborough adaptation of the Tower of London (S‐TOL) using near‐infrared spectroscopy. Frontiers in Human Neuroscience, 8, 185 10.3389/fnhum.2014.00185 24734017 PMC3975118

[brb31789-bib-0069] Sartor, C. E. , McCutcheon, V. V. , Pommer, N. E. , Nelson, E. C. , Grant, J. D. , Duncan, A. E. , … Heath, A. C. (2011). Common genetic and environmental contributions to post‐traumatic stress disorder and alcohol dependence in young women. Psychological Medicine, 41(7), 1497–1505. 10.1017/S0033291710002072 21054919 PMC3377473

[brb31789-bib-0070] Shallice, T. (1982). Specific impairments of planning. Philosophical Transactions of the Royal Society of London. Series B, Biological Sciences, 298(1089), 199–209. 10.1098/rstb.1982.0082 6125971

[brb31789-bib-0071] Sheerin, C. M. , Bountress, K. E. , Meyers, J. L. , Saenz de Viteri, S. S. , Shen, H. , Maihofer, A. X. , … Amstadter, A. B. (2020). Shared molecular genetic risk of alcohol dependence and posttraumatic stress disorder (PTSD). Psychology of Addictive Behaviors, 10.1037/adb0000568 PMC7394716 32191043

[brb31789-bib-0072] Springer, K. W. , Sheridan, J. , Kuo, D. , & Carnes, M. (2007). Long‐term physical and mental health consequences of childhood physical abuse: Results from a large population‐based sample of men and women. Child Abuse and Neglect, 31(5), 517–530. 10.1016/j.chiabu.2007.01.003 17532465 PMC3031095

[brb31789-bib-0073] Stein, M. B. , Jang, K. L. , Taylor, S. , Vernon, P. A. , & Livesley, W. J. (2002). Genetic and environmental influences on trauma exposure and posttraumatic stress disorder symptoms: A twin study. American Journal of Psychiatry, 159(10), 1675–1681. 10.1176/appi.ajp.159.10.1675 12359672

[brb31789-bib-0074] Tripp, J. C. , McDevitt‐Murphy, M. E. , Avery, M. L. , & Bracken, K. L. (2015). PTSD Symptoms, emotion dysregulation, and alcohol‐related consequences among college students with a trauma history. Journal of Dual Diagnosis, 11(2), 107–117. 10.1080/15504263.2015.1025013 25793550 PMC4437848

[brb31789-bib-0075] True, W. R. , Rice, J. , Eisen, S. A. , Heath, A. C. , Goldberg, J. , Lyons, M. J. , & Nowak, J. (1993). A twin study of genetic and environmental contributions to liability for posttraumatic stress symptoms. Archives of General Psychiatry, 50(4), 257–264. 10.1001/archpsyc.1993.01820160019002 8466386

[brb31789-bib-0076] Twamley, E. W. , Allard, C. B. , Thorp, S. R. , Norman, S. B. , Hami cissell, S. , Hughes berardi, K. , … Stein, M. B. (2009). Cognitive impairment and functioning in PTSD related to intimate partner violence. Journal of the International Neuropsychological Society, 15(6), 879–887. 10.1017/S135561770999049X 19703324

[brb31789-bib-0077] Vasterling, J. J. , Duke, L. M. , Brailey, K. , Constans, J. I. , Allain, A. N. Jr , & Sutker, P. B. (2002). Attention, learning, and memory performances and intellectual resources in Vietnam veterans: PTSD and no disorder comparisons. Neuropsychology, 16(1), 5–14. 10.1037//0894-4105.16.1.5 11853357

[brb31789-bib-0078] Walters, R. K. , Polimanti, R. , Johnson, E. C. , McClintick, J. N. , Adams, M. J. , Adkins, A. E. , … Agrawal, A. (2018). Transancestral GWAS of alcohol dependence reveals common genetic underpinnings with psychiatric disorders. Nature Neuroscience, 21(12), 1656–1669. 10.1038/s41593-018-0275-1 30482948 PMC6430207

